# Supplementary material for: Chitosan Grafted with Thermoresponsive Poly(di(ethylene glycol) Methyl Ether Methacrylate) for Cell Culture Applications
Source: Polymers (Basel). 2023 Mar 18;15(6):1515. doi: 10.3390/polym15061515 (PMC10051194; doi:10.3390/polym15061515)
Supplement: Supplementary file 1 [file polymers-15-01515-s001.zip › polymers-2288726-supplementary.pdf]

polymers

Supporting Information

Chitosan Grafted with Thermoresponsive Poly(di(ethylene glycol) Methyl Ether Methacrylate) for Cell Culture Applications

Natun Dasgupta, Duo Sun, Maud Gorbet, and Mario Gauthier*

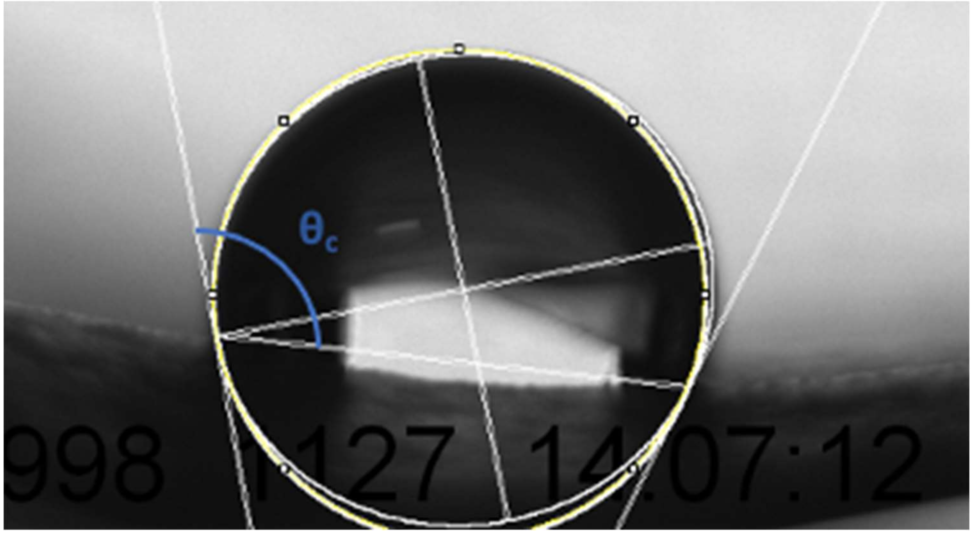

Figure S1: Sessile-drop contact angle (θ_c) determination using ImageJ.

Table S1: Grafting efficiency of PMEO$_2$MA to chitosan using Chito-RAFT agent.

Sample	Trial	Chito-RAFT agent (W_i, mg)	MEO$_2$MA (W_m, mg)	Recovered (W_r, mg)	Average grafting efficiency (%)	Average PMEO$_2$MA content (wt%)
**Chito-*g*-PMEO$_2$MA (15 wt%)**	1	110	19.5	120		
	2	113	19.9	123	49±2	8.7±1
	3	112	19.7	121		
**Chito-*g*-PMEO$_2$MA (30 wt%)**	1	110	47.3	132		
	2	110	47.3	134	47±3	20.3±1
	3	113	48.4	134		
**Chito-*g*-PMEO$_2$MA (60 wt%)**	1	110	166	152		
	2	110	166	161	29±3	43±3
	3	112	168	162		

Table S2: Comparison of the apparent number-average molecular weights (M_n) obtained by GPC analysis of the cleaved PMEO$_2$MA chains.

**Cleaved Samples**	**M_n from GPC (kg/mol)**	**PDI**
Chito-*g*-PMEO$_2$MA (15 wt%)	43	1.82
Chito-*g*-PMEO$_2$MA (30 wt%)	85	1.71
Chito-*g*-PMEO$_2$MA (60 wt%)	128	1.80
Chito-*g*-PMEO$_2$MA (60 wt%, High DS)	105	1.88

Table S3: Contact angle (in degrees) from t = 0 to 4 s for selected samples at 22 and 40 ºC.

**Time (s)**	**$PMEO_2MA$ on glass slide**		**SNP-*g*-$PMEO_2MA$ (15 wt%)**		**SNP-*g*-$PMEO_2MA$ (30 wt%)**		**SNP-*g*-$PMEO_2MA$ (60 wt%)**	
	**@22 ºC**	**@40 ºC**	**@22 ºC**	**@40 ºC**	**@22 ºC**	**@40 ºC**	**@22 ºC**	**@40 ºC**
**0**	73±2	88±1	75±3	103.7±2	95±2	109±1.5	91±1.3	102±1.2
**1**	70.4±1.7	88.7±1.2	66±4.8	102.9±3.4	92.4±2	107±2	92.5±1	100.5±2
**2**	72±2	89.2±1.5	62.7±5	103.5±4.7	86.7±4	106.8±2	91±1.2	102±2.7
**3**	73.6±1.5	85.1±2	62.2±4.3	102.3±4.9	81.7±4.7	106.9±2.1	93.2±2	101±3
**4**	74.8±2.7	85.6±2.6	60.2±6	104.8±3.7	83.3±3.6	106.6±1.7	92.1±3	102±4.2

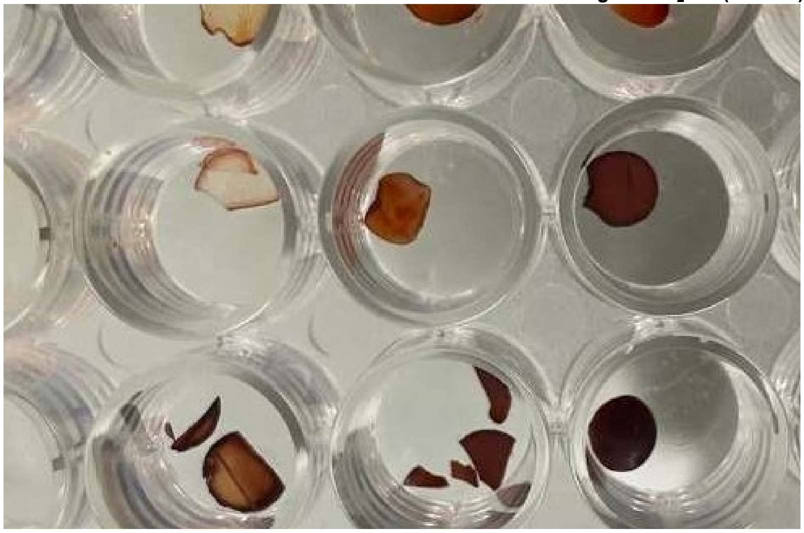

Figure S2: Sorption of the dye on the films.

Table S4: Water uptake index (WUI) of selected chitosan films at 22 °C

Sample	Trial	Initial Weight (W_d, mg)	After Water Uptake (2 h) (W_w, mg)	Water Uptake Index (WUI)	Average WUI (%)
**Chitosan**	1	24.7	65.2	164	
	2	40.7	110.5	171	169±5
	3	36.7	100.1	173	
**Chito-RAFT**	1	33.2	79	138	
	2	28.2	81	187	154±29
	3	42	99.4	137	
**Chito-*g*-PMEO$_2$MA (15 wt%)**	1	45.3	122.4	170	
	2	34.7	83.8	141	151±17
	3	30.7	74	141	
**Chito-*g*-PMEO$_2$MA (30 wt%)**	1	39.4	71.5	81	
	2	34.4	65.1	89	85±4
	3	42.1	77	83	
**Chito-*g*-PMEO$_2$MA (60 wt%)**	1	34.2	60	75	
	2	45	75.1	67	78±12
	3	33	63	91	

Table S5: Water uptake Index (WUI) of selected chitosan films at 37 ^{o}C

Sample	Trial	Initial Weight (W_d, mg)	After Water Uptake (2 h) (W_w, mg)	Water Uptake Index (WUI)	Average WUI (%)
**Chitosan**	1	35.5	89.8	153	
	2	46.2	138	199	158±39
	3	41.8	92.5	121	
**Chito-RAFT**	1	29.7	73.7	148	
	2	30.5	66.2	117	142±23
	3	30.3	79.1	161	
**Chito-*g*-PMEO$_2$MA (15 wt%)**	1	36.2	83.4	130	
	2	27.8	57.7	108	117±12
	3	24.6	52.2	112	
**Chito-*g*-PMEO$_2$MA (30 wt%)**	1	27.6	43.4	57	
	2	40.1	65.8	64	61±3
	3	29.5	47.6	62	
**Chito-*g*-PMEO$_2$MA (60 wt%)**	1	28.5	45	58	
	2	37.5	50	33	55±20
	3	27.5	47.5	73	
